# Amyloid in the islets of Langerhans: Thoughts and some historical aspects

**DOI:** 10.3109/03009734.2011.573884

**Published:** 2011-04-12

**Authors:** Per Westermark

**Affiliations:** Department of Immunology, Genetics and Pathology, Uppsala University, Uppsala, Sweden

**Keywords:** Amyloid, β-cell mass, β-cells, IAPP, islets of Langerhans, protein aggregation, type 2 diabetes

## Abstract

Deposition of amyloid, derived from the polypeptide hormone islet amyloid polypeptide (IAPP; ‘amylin’) is the single most typical islet alteration in type 2 diabetes. Islet amyloid was described as hyalinization already in 1901, but not until 1986 was it understood that it is a polymerization product of a novel β-cell regulatory product. The subject of this focused review deals with the pathogenesis and importance of the islet amyloid itself, not with the biological effect of the polypeptide. Similar to the situation in Alzheimer's disease, it has been argued that the amyloid may not be of importance since there is no strict correlation between the degree of islet amyloid infiltration and the disease. However, it is hardly discussable that the amyloid is important in subjects where islets have been destroyed by pronounced islet amyloid deposits. Even when there is less islet amyloid the deposits are widely spread, and β-cells show ultrastructural signs of cell membrane destruction. It is suggested that type 2 diabetes is heterogeneous and that in one major subtype aggregation of IAPP into amyloid fibrils is determining the progressive loss of β-cells. Interestingly, development of islet amyloid may be an important event in the loss of β-cell function after islet transplantation into type 1 diabetic subjects.

## Introduction

It is now 25 years since we elucidated the nature of the amyloid in the islets of Langerhans (1). Deposition of amyloid is the most characteristic alteration in the islets of Langerhans in type 2 diabetes and was described already 110 years ago (2,3); it was for long time named hyalinization of the islets. A resemblance to amyloid was noted at an early date, and when the nature of this substance was debated the name ‘para-amyloid’ was sometimes used for deposits like those in the islets (4). However, not until the studies by Ehrlich and Ratner (5) was the material accepted as a ‘real’ form of amyloid. It may be mentioned that the discussion about inclusion criteria for amyloid is still on-going (6). For a long time, the interest for this alteration, which is quite characteristic for type 2 diabetes, was generally low, but it was an enigma mainly for pathologists who noticed the islet alteration when examining autopsy specimens. The low interest in amyloid among researchers in the diabetes field might have been due to the fact that islet amyloid is missing in mouse and rat models of diabetes. The reason for this absence became obvious soon after our description of the nature of human (and feline) islet amyloid (1,7). Another reason for the lack of interest is that islet amyloid is not solely seen in association with diabetes; it occurs also in non-diabetic subjects but less commonly and to a lower degree (8–10). The finding that islet amyloid is composed by a previously unknown polypeptide hormone immediately increased the interest in islet amyloid.

**Figure:**
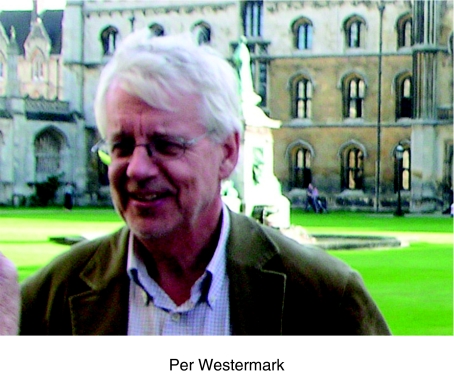
**Winner of the Rudbeck Award, 2010 at the Medical Faculty of Uppsala University.For his pioneering research on the mechanisms of islet amyloidosis and its impact on the pathogenesis of type 2 diabetes.**

## The elucidation of the islet amyloid nature

**Figure 1.**
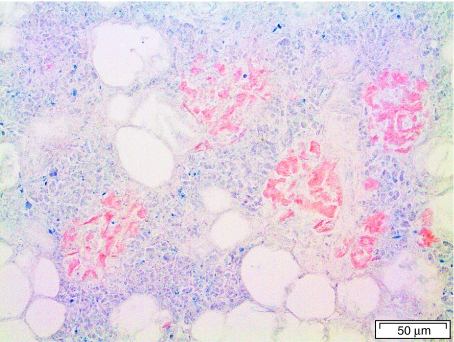
Pancreatic section of case IsN13 with islets filled with amyloid, stained with Congo red. This material was used to purify the IAPP giving the first amino acid sequence from human islet origin. There are signs of pronounced autolysis, which did not affect the quality of the purified peptide. There was also partial exocrine atrophy which was helpful in that it made the amyloid more concentrated.

Gellerstedt, a professor of pathology at Uppsala University, noted already in 1938 that the Congo red staining property of islet amyloid was slightly different from that of systemic amyloid (4). Also histochemical studies indicated that the islet amyloid was different (11,12). However, only an amino acid sequence analysis could determine the nature of the material. It was not an easy task to extract, purify, and determine the nature of the main protein in islet amyloid. In 1971 the two first fibril proteins from systemic amyloidosis had been purified and determined by Edman degradation. One was found to be of immunoglobulin light chain origin (13), while the second turned out to be a previously unknown protein, now called AA (14). The third amyloid fibril protein to be characterized was calcitonin (or procalcitonin) in the C-cell tumor thyroid medullary carcinoma (15). In all these instances, the tissue material serving for protein purification was very rich in amyloid, making identification of the main protein comparatively easy. Dealing with amyloid in the islets of Langerhans was a completely different problem. This amyloid is strictly limited to the around 1 million islets which constitute about 1% of the total pancreatic mass. A second problem was the unusual insolubility of this amyloid (12). Previously studied amyloid proteins had been purified after solubilization in guanidine hydrochloride, but islet amyloid turned out to be completely insoluble in this solvent. After years of trials, the solution to the problem came with a β-cell tumor (insulinoma), rich in amyloid. With this material a new approach was used, including solubilization in concentrated formic acid, used for identification of the Aβ protein (16), a new purification method with the aid of high-performance liquid chromatography and access to a new sensitive gas phase sequenator. In this way, the nature of the protein was finally elucidated (1). Surprisingly, it turned out to be a novel β-cell protein, not related to insulin or its precursor. It was initially named insulinoma (or islet) amyloid peptide (IAP), which soon changed to islet amyloid polypeptide (IAPP) since the abbreviation IAP was already used. Further analyses, also of protein purified from amyloid derived from human and cat islets (Figure 1), revealed a 37-amino acid residue polypeptide belonging to the calcitonin gene-related peptide (CGRP) family (7,17). Soon afterwards, our findings were verified by another research group (18). Parts of this group later named the peptide ‘amylin’ (19). By immunohistochemistry (7,20), immune electron microscopy (21,22), and later *in-situ* hybridization (23) it was shown that IAPP is a product of islet β-cells (24). IAPP is stored together with insulin in the secretory vesicles. It is located to the halo region where also proinsulin and C-peptide are located. Although most of the circulating IAPP is derived from islet β-cells, there is IAPP expression in some gastrointestinal endocrine cells, in certain peripheral ganglia, and in the brain (for review, see (25)).

### IAPP in animal species

**Figure 2.**
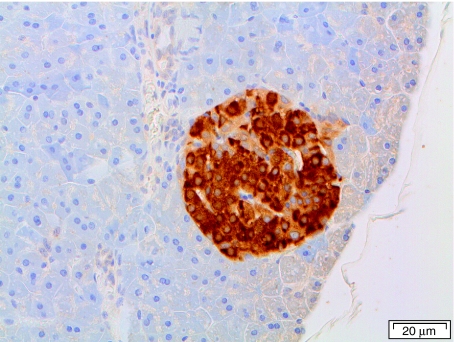
Pancreatic section of a common shrew (*Sorex araneus*), labeled with antibodies against IAPP. There is a strong reaction with the β-cells, which constitute the majority of islet cells.

IAPP is a conserved molecule and is expressed in mammals (26,27) (Figure 2), birds (28), fishes (29,30), and reptiles (Westermark and Westermark, unpublished) although islet amyloid is seen only in a limited number of species. As mentioned in the introduction, islet amyloid does not appear in mouse or rat islets. It is, however, a typical alteration in diabetes in many non-human primate species (31,32) as well as in cat species (33–35). Structural studies of islet amyloid in several mammalian species revealed that IAPP is a strongly conserved molecule but that there are considerable species variations in a middle segment of the peptide. It turned out that mouse and rat IAPP, which are identical, carry three proline residues in the 20–29 segment, where the human molecule has none and the cat has one (26,36). While a synthetic peptide corresponding to human IAPP 20–29 is extremely fibrillogenic *in vitro*, that corresponding to the rat/mouse molecule is not (37). Consequently, it turned out that the reason why islet amyloid is not found in these rodents is simply that their IAPP is not amyloidogenic. Parenthetically, it may be mentioned that these species discrepancies have been utilized by a pharmaceutical company to make a non-fibrillogenic IAPP variant, used for treatment of type 1 and type 2 diabetes.

### Amyloid in the degu: an interesting exception

**Figure 3.**
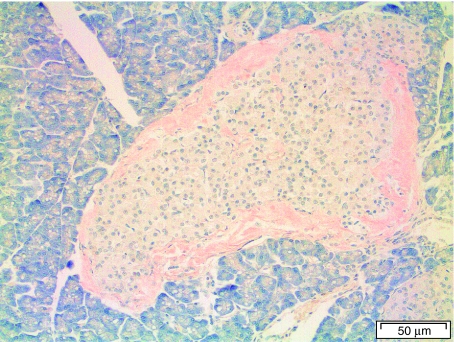
Pancreatic section of a degu (*Octodon degus*) with heavy amyloid deposits at the islet periphery, stained with Congo red.

There is a remarkable exception from the rule that islet amyloid does not occur in species with apparently non-fibrillogenic IAPP, and that is the South American hystricomorphous rodent degu (*Octodon degus*). This animal is prone to develop diabetes in captivity (38). Degu IAPP has proline residues at positions 28 and 29 (39). In spite of this, islet amyloid is common in elderly and diabetic animals (Figure 3). Direct amino acid sequence analysis of purified degu islet amyloid revealed surprisingly that the fibril protein in this species is derived from insulin (40). In a way, this finding closed the circle, since insulin was, for natural reasons, believed to be the fibril protein in human islet amyloid (41) before IAPP was discovered. Insulin is an amyloid protein also in human, although an iatrogenic one; amyloid ‘tumors’ may appear at the site of repeated insulin injections in type 1 diabetic subjects (42,43).

## Morphology of islet amyloid

**Figure 4.**
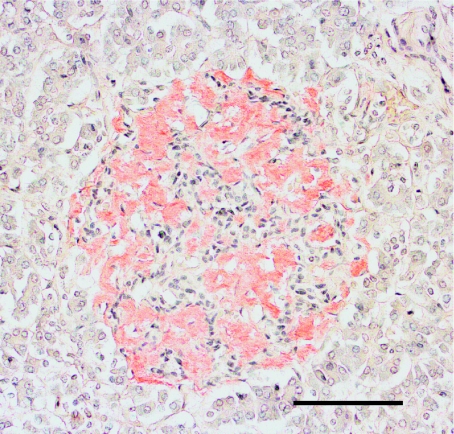
Human islet from a diabetic subject. Most of the islet has been converted into amyloid, but there are still cords of cells, a majority being β-cells, most probably dysfunctional. Congo red. Bar: 50 μm.

**Figure 5.**
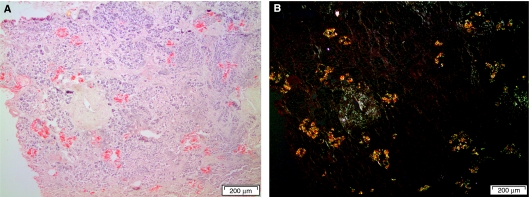
Section from the pancreas of a diabetic subject with severe islet infiltration of amyloid in all islets, visualized with Congo red. The section in A is seen in ordinary light, while in B polarized light with crossed polars has been used. A bright yellow birefringence is evident.

**Figure 6.**
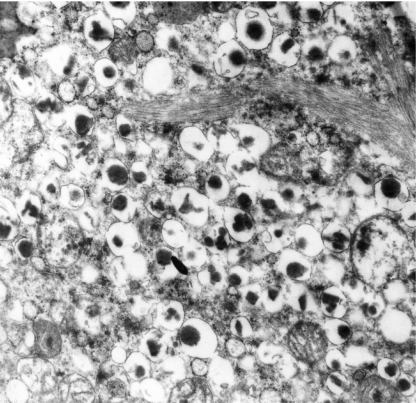
Electron micrograph showing a part of a β-cell from a diabetic subject. There are bundles of typical amyloid fibrils which penetrate deeply into the cells. Note that the cell is filled with characteristic insulin granules.

Islet amyloid is restricted to the islet area and is not found systemically or in other parts of the pancreas. In human, islet amyloid is extracellular, and it has been difficult to find certain intracellular amyloid. When the deposits are small, amyloid can be seen as thin layers between endocrine cells and capillaries and are tapestrying the outside of the islet. When larger, the deposits can form large masses completely remodeling the islets (Figure 4). Amyloid can be visualized with ordinary amyloid dyes such as Congo red and shows the typical birefringence that can vary between yellow and green (Figure 5), although erroneously often referred to as ‘apple green’ (44). It should be noted that the affinity of this kind of amyloid for Congo red often is weak or very weak, making it easy to underestimate the amount of deposited material or even to totally miss the amyloid. By definition, islet amyloid consists of thin fibrils, around 10 nm in width, but with an undetermined length. Within the deposits, the fibrils are not organized, but close to β-cells there is often an orientation of bundles of fibrils towards the cell membrane (24). Typically, bundles of fibrils run into deep pockets of β-cells, seemingly penetrating the cell membrane (Figure 6). A conspicuous feature of β-cells close to islet amyloid is the distortion of the architecture of the outer part of the cells and loss of an evident basement membrane (24). The lack of intracellular amyloid in β-cells of diabetic individuals does not necessarily mean that aggregation of IAPP to fibrils may not take place here. In models with more rapidly developing islet amyloid, e.g. in human islets transplanted into nude mice or in islets cultured *in vitro*, small amyloid deposits develop within a few days or weeks (45,46). This very first amyloid is intracellular. We have suggested that this first amyloid leads to apoptosis of the cell, leaving the amyloid, which is resistant to degradation, extracellularly where it seeds further amyloid formation from exocytosed IAPP (46). Such an event might explain the deep pockets into β-cells formed by the fibrils when these are elongated by addition of new IAPP molecules at the cellular side (47).

### The spreading of islet amyloid

An interesting phenomenon is that even very small amyloid deposits are widely spread throughout an individual islet (10), although other islets in the neighbourhood are free. This might indicate that the abnormality has affected all cells in the islet. Amyloidogenesis is a nucleation-dependent phenomenon and does not start until a nucleus has formed (48). However, when the first fibrils have assembled, the process moves on rapidly as long as the peptide is present above a critical concentration. The widely spread amyloid in single islets may simply reflect such events. Whether or not islets may communicate so that amyloid can spread from one islet to another is still an open question.

## Why does IAPP form amyloid in type 2 diabetes?

The pathogenesis of any localized form of amyloid is not clear. Although most human proteins contain segments that in theory are able to assemble into amyloid fibrils, most such parts are hidden in the molecules and may be exposed only after cleavage (49). In the case of IAPP, aberrant cleavage is not necessary for fibrillogenesis since only full-length molecules have been identified in the deposits, although small amounts of propeptides can be demonstrated by immunological techniques (50,51). Still, aberration in the cleavage of pro-IAPP as an initial step in amyloidogenesis cannot be ruled out (52). An increased local concentration of the fibrillogenic protein may be important. Over-production of insulin, often seen in type 2 diabetes as a response to insulin resistance, is associated with increased release also of IAPP since these two β-cell products are normally regulated in parallel (53). IAPP is a very fibrillogenic peptide *in vitro*, and its native conformation must be controlled *in vivo*. Consequently, a further possible factor in amyloidogenesis might be a dysregulation of chaperones, normally hindering the strongly fibrillogenic molecule IAPP from making amyloid. It is not known exactly how IAPP is stored in granules and which factors normally inhibit fibril formation, but, experimentally, insulin is a strong inhibitor (54–56). Although insulin is mainly stored in crystalline form in the granule core and IAPP in the halo region, it is possible that there is enough insulin in the halo to inhibit fibril formation. Proinsulin, present in the halo, is also an inhibitor (54). Finally, there may be unknown inhibitors and promoters in IAPP amyloidogenesis. Among additional components present in all forms of amyloid and which may affect amyloidogenesis are heparan sulfate (57) and serum amyloid P-component (SAP) (58,59).

## Is islet amyloid of any importance in type 2 diabetes?

This question can be rephrased as to whether aggregated IAPP in any form is pathogenically important since there is evidence that not fully developed amyloid fibrils but smaller, oligomeric assemblies exert toxic effects on β-cells and thereby kill them (60). It has even been suggested that the amyloid itself is innocent and that its formation rather is a protection mechanism by which the dangerous oligomeric species are taken care of (61). In this paper, I will not discuss oligomers more, and interested readers are referred to a recent review (25). However, when looking at a pancreatic section from a patient with type 2 diabetes where almost all of the islets are converted to amyloid (Figure 4), it is very difficult to believe that the amyloid itself is without importance. Both insulin (62) and IAPP(63) are normally released in pulses. It is tempting to believe that the tightly regulated pulses are disturbed by the amyloid interposed between β-cells and capillaries and most probably interfering with the cell membrane function.

Another important issue is whether formation of islet amyloid is a pathogenic event in the development of type 2 diabetes or just participating in the final destruction of islets. This question cannot be answered presently (64). A reason why islet amyloid has been denied as a pathogenic mechanism in diabetogenesis is that islet amyloid does not occur in all individuals with type 2 diabetes. Interestingly, in a recent study on a baboon colony, islet amyloid appeared before the development of diabetes, and the amount of amyloid correlated well with the progression of the disease (65). It has not been possible to demonstrate such a direct association in human. What we call type 2 diabetes is heterogeneous in human, and most probably several different mechanisms act in the pathogenesis. It is quite possible that, in one major subgroup of individuals, aggregation of IAPP and development of islet amyloid actually is of major importance. It is also possible that deposition of islet amyloid acts in concert with other pathogenic mechanisms. This possibility will probably not be answered until methods have been developed by which amyloid deposits can be demonstrated *in vivo*, e.g. by positron emission tomography (PET), or until methods to prevent islet amyloid formation or even to dissolve already formed fibrils have been introduced.

## The β-cell mass in type 2 diabetes

For a long time there was no agreement concerning the question whether there is a reduction in islet volume and β-cell mass in type 2 diabetes. In early studies, types 1 and 2 diabetes were usually not clearly separated (66,67). Several later studies have shown a modest reduction of the islet mass in type 2 diabetes compared to age-matched controls, but there has been a considerable overlap (68,69). This reduction depends mainly on a smaller β-cell mass, shown in a number of studies (64,70–72).

In one study we measured the total pancreatic volume as well as islet volume in 12 subjects with type 2 diabetes and 15 age-matched controls (69). The total islet volume in non-diabetic subjects was 1.60 ± 0.16cm^3^ (mean ± SEM) and in diabetic individuals 1.01 ± 0.12 cm^3^ (*P* < 0.01). In another study we found that the percentage of β-cells was significantly lower in islets of type 2 diabetic individuals compared to non-diabetic controls (43.0% ± 22% versus 59.2% ± 1.9%; *P* < 0.001) (70). If we assume that the materials in these two studies, emerging from the same department, are comparable, an estimate of β-cell volume in type 2 diabetes and controls would be 0.43 ± 0.05 and 0.95 ± 0.09 cm^3^, respectively. In reality, the difference should be even greater since in this calculation the volume taken up by amyloid has not been taken into consideration. These data fit well with a finding that the β-cell area in diabetic subjects was reduced only in amyloid-containing islets (64). Even more intriguing was the significant difference in β-cell percentage between islets in non-diabetic subjects with amyloid and those without any deposits (70). While islets in those without any amyloid contained 64.6% ± 1.4% β-cells, the percentage of β-cells in islets in individuals with islet amyloid was 55.9% ± 2.5% (*P* < 0.01). This indicates that deposition of islet amyloid is directly associated with β-cell loss and not only a result of the diabetic state *per se*.

### Limitations of islet volume determination in human

All islet volume determinations suffer from the limitation that they have had to be performed on autopsy material. That means that individuals with diabetes usually have had their disease for several years, while it would have been of greater interest to study the pancreas very early in the diabetic state, or preferably before diabetes had become established. This is presently impossible in humans until new methods have been developed. Since the pancreas is very sensitive to autolytic changes, obscuring histological details, it should be pointed out that studies have to be performed on material obtained well within 24 hours after death, something that was possible in the 1970s but is completely impossible today, at least in Sweden although it seems possible in some other countries (72).

There are some good animal models which should be relevant for human type 2 diabetes. Thus, Howard showed that the diabetes which develops in *Macaca nigra* resembles the human form (31). Islet amyloid with loss of β-cells developed before overt diabetes. A type 2-like diabetes can also be found in elderly domestic cats (34,73). Baboons may also offer a relevant model (see above). In addition, there are transgenic mouse and rat models over-expressing human IAPP in which diabetes associated with islet amyloid develops (for review, see (25)).

### Functional β-cell mass

Even in islets filled with amyloid, there are a number of β-cells filled with secretory vesicles (24). These cells are in direct contact with amyloid fibrils and show morphological signs of membrane damage and are most probably not functioning in a normal way. Therefore, the functional β-cell mass may be very different from the total β-cell mass. A morphological measurement of the total β-cell mass does not give true functional mass.

## Amyloid in transplanted human islets

Human islets isolated from organ donors may be transplanted to diabetic recipients, particularly in cases with diabetes which is difficult to regulate with the common regimens. Most commonly this is done via an islet infusion into the portal vein. Initially, many patients become independent of exogeneous insulin injections, but most commonly the function of the grafted islets deteriorates with time, and after a few years most individuals need insulin treatment (recently reviewed in (74)). Pioneered by Andersson (75), human islets can be isolated and transplanted into nude mice, some of which were made diabetic by means of alloxan injections. We found that normal human islets, transplanted under the kidney capsule (45) or into the spleen or liver (76), rapidly develop amyloid deposits. We therefore questioned whether amyloid may form also in clinically transplanted islets (77). Recently, we had the opportunity to study the liver of one patient who had been transplanted 5 years earlier and who died from a cardiac infarction. Indeed, almost 50% of the studied islets contained IAPP amyloid, often in a substantial amount (78). This finding has recently been verified by studies of additional cases (Westermark et al., unpublished). Therefore, by transplantation of normal human islets into type 1 diabetic individuals, type 2 diabetic alterations are induced in these islets.

## Conclusion

Porte pointed out two findings in type 2 diabetes, providing clues to the pathogenesis: the relative hyperproinsulinemia and the deposits of amyloid in the islets (79). Hyperproinsulinemia would most likely be associated with aberrant cleavage also of pro-IAPP. There is evidence that the aberrant cleavage product may start amyloid deposition by the formation of a nucleus (46,52). The amyloid formed may cause β-cell apoptosis and dysfunction of remaining cells. These mechanisms may also be important for the loss of function of transplanted human islets.
